# Quality of Life in Underrepresented Cancer Populations

**DOI:** 10.3390/cancers14143417

**Published:** 2022-07-14

**Authors:** Gwendolyn P. Quinn, Matthew B. Schabath

**Affiliations:** 1 Department of Obstetrics and Gynecology, Grossman School of Medicine, New York, NY 10016, USA; 2 Department of Cancer Epidemiology, H. Lee Moffitt Cancer Center & Research Institute, Tampa, FL 33612, USA; matthew.schabath@moffitt.org

This series of six articles (five original articles and one review) is presented by international leaders in health disparities research. Marginalized and underrepresented populations comprise a broad array of communities that include racial and ethnic minorities, adolescents and young adults, older adults, persons living in rural areas, and sexual and gender minorities (i.e., LGBTQ+ populations). All people, regardless of their demographics, desire and deserve quality of life throughout the cancer care continuum, from cancer treatment to survivorship. Quality of life in a broad sense encompasses one’s sense of well-being and ability to carry out daily activities [1]. However, discrete aspects of life quality involve a host of constructs: financial, being free from pain, sexual health, fertility, access to care, time spent in seeking care, and supportive and palliative care services.

This Special Issue focuses on aspects of quality of life in underrepresented populations and highlights the variety of experiences within these populations. Dos Santos et al. [2] examined senior adult cancer patients, aged over 70, regarding chemotherapy-induced cognitive impairment. Their results suggest that preexisting conditions such as malnutrition and preexisting cognition issues exacerbated cognitive impairment post chemotherapy compared with senior adults without these preexisting conditions. These results underscore the need to assess older adults’ physical and mental health status prior to treatment to achieve the best outcomes in survivorship. Prior research has suggested that fatigue [3] may be worse in adolescent and young adult (AYA) patients due to developmental changes and the need for longer durations of sleep. The interplay of young age and a cancer diagnosis likely creates an even more elevated risk for excessive fatigue. To address this, Fauske et al. [4] developed an intervention to improve chronic fatigue in AYA. Their pilot study showed significant improvement in reducing fatigue and improving energy levels. This study underscores the need to develop interventions that are tailored to underserved groups during the oncology care experience rather than assuming a one-size-fits-all approach. 

Another unique aspect of AYA is that they are still in their reproductive years, in contrast to cancer patients diagnosed at the median the age of ~66 [5]. Cancer treatment has potential for a deleterious effect on fertility, and a loss of fertility or infertility is often cited as being as horrific as the cancer diagnosis. Multiple professional organizations (ASCO, AAP, ASRM) cite the need for fertility counseling and referral for all AYA, yet multiple constraints such as need for immediate treatment, no access to reproductive specialists, and lack of clinician training can hinder discussions. Theroux et al. [6] examined decision quality among adolescent males with regard to sperm-banking. These results showed that the offer of counsel and the use of a decision tool improved decision quality among males who attempted to bank sperm and those who did not. Furthermore, among those who did not attempt to bank their sperm, 40% of respondents thought they may regret their decision in the future, and no males who did bank their sperm predicted that they would regret this decision. This study highlights the need for patient-facing oncology tools that can improve decision quality, which may lead to improved satisfaction in cancer survivorship. 

Goncalves [7] conducted a narrative review of the offer of fertility-preservation counsel and referral for females with a cancer diagnosis. Findings suggest that among females who received counsel for fertility preservation, decisional regret was low whether preservation was pursued or not. Similar to the study among males by Theroux et al. [6], this study showcases the need for informed decision making regarding fertility preservation in AYA with a cancer diagnosis.

Rurality has played key role in access to quality oncology care and the ability to adhere to treatment. Kano et al. [8] conducted a survey study among breast and gynecologic cancer patients living in rural areas concerning their barriers to and facilitators for receiving cancer care. Results revealed that, on average, patients traveled over 30 miles to receive cancer care and 78% had prior comorbid conditions requiring care outside of the cancer center. Furthermore, 46% of those who had completed treatment did not have a survivorship care plan. Patients living in rural areas not only must travel far distances to receive their care but must also seek healthcare elsewhere for conditions not related to their cancer care, creating a significant amount of travel and time spent in maintaining health. 

LGBTQ+ patients, or sexual and gender minorities, can have unique psychosocial care needs during their cancer care experiences. There are a numerous studies documenting worse outcomes for LGBTQ+ people in the oncology domain (reviewed in [9]). However, Borowczak et al. [10] conducted a small qualitative pilot study comparing the survivorship experiences of lesbian and heterosexual women with a breast cancer diagnosis. Findings revealed that lesbian women had higher satisfaction with their cancer care experiences and a more positive self-image than heterosexual women. A majority of women in both groups chose to undergo breast reconstruction. While the sample size for the study was small, it is important in that it identifies that sexual minority women may experience social support and their cancer care through a different perspective than straight women. Cumulatively, the studies in this Special Issue highlight an array of special populations related to age, geography, and sexual orientation and their unique psychosocial care needs. Future interventions should consider the need to tailor strategies for underserved and minority populations during the cancer care trajectory.
